# EvaLuation of early CRRT and beta-blocker InTervention in patients with ECMO (ELITE) trial: study protocol for a 2 × 2 partial factorial randomized controlled trial

**DOI:** 10.1186/s13063-022-06617-x

**Published:** 2022-08-19

**Authors:** Xiaofang Wang, Hong Wang, Xin Du, Zhiyan Wang, Chenglong Li, Craig S. Anderson, Jinying Zhang, Xiaotong Hou, Jianzeng Dong

**Affiliations:** 1 Department of Cardiology, The First Affiliated Hospital of Zhengzhou University, Zhengzhou, Henan China; 2 Center for Cardiac Intensive Care, Beijing Anzhen Hospital, Capital Medical University, 2# Anzhen Road, Chaoyang District, Beijing, 100029 China; 3 Department of Cardiology, Beijing Anzhen Hospital, Capital Medical University, 2# Anzhen Road, Chaoyang District, Beijing, 100029 China; 4 Heart Health Research Center, Beijing, China; 5 George Institute for Global Health, Faculty of Medicine, University of New South Wales, Sydney, Australia

**Keywords:** Cardiogenic shock, Extracorporeal membrane oxygenation, Acute kidney injury, Renal replacement therapy, Mortality, Randomization

## Abstract

**Background:**

In critically ill patients requiring extracorporeal membrane oxygenation (ECMO) therapy, early initiation of continuous renal replacement therapy (CRRT) and beta-blockade of catecholamine-induced inotropic effects may improve outcomes.

**Methods:**

A 2 × 2 partial factorial randomized controlled trial in eligible ECMO patients without a clear indication or contraindication to either intervention is centrally randomly assigned to (A) early or conventional-indicated CRRT and/or (B) beta-blocker or usual care. The primary outcome is all-cause mortality at 30 days for both arms. A total of 496 participants provides 80% power to determine a 20% risk reduction in mortality at 30 days with 5% type I error.

**Discussion:**

This trial will help define the role of early CRRT and beta-blockade in ECMO patients. There have been 89 patients enrolled at 10 hospitals in study A and is ongoing. However, study B was stopped in August 2019 in the absence of any patients being enrolled.

**Trial registration:**

ClinicalTrials.govNCT03549923. Registered on 8 June 2018. World Health Organization International Clinical Trials Registry Platform (WHO ICTEP) network. The Ethics Committee of Beijing Anzhen Hospital Approval ID is 2018013.

## Background

### Introduction

Veno-arterial extracorporeal membrane oxygenation (VA-ECMO) provides short-term circulatory support for critically ill patients with severe cardiac shock, but mortality remains high (60 to 75%) [1] and acute kidney injury (AKI) and fluid overload (FO) are frequent complications [2, 3] that are managed with continuous renal replacement therapy (CRRT) [3–6]. However, the optimal timing of CRRT is uncertain: the positive results of the early vs late initiation of renal replacement therapy in critically ill patients with acute kidney injury (the ELAIN) trial [7] were not confirmed in another study, the Artificial Kidney Initiation in Kidney Injury (AKIKI) [8]. As critically ill patients have excessive sympathetic activation and autonomic dysfunction from high circulating catecholamines that may compromise cardiac function [9–14], beta-blockade could improve outcomes by improving myocardial oxygen consumption, ventricular remodeling, and left ventricular function [15, 16]. A randomized, open, single-center study in patients with septic shock requiring norepinephrine to maintain mean arterial pressure showed a benefit of esmolol on 28-day mortality [17]. The circulatory support offered by ECMO may avoid the negative inotropic effect of beta-blockers in the setting of cardiogenic shock [18]. We designed the EvaLuation of early CRRT and beta-blocker InTervention in patients with ECMO (ELITE) trial to determine the effects of early CRRT versus conventionally indicated CRRT and/or beta-blockade on top of routine care.

### Objective

The objective is to determine whether (A) early CRRT compared to conventional-indicated CRRT and (B) beta-blockade with esmolol compared to standard treatment will reduce 30-day mortality in ECMO patients.

## Methods

### Study design (Fig. 1)

ELITE is a prospective, multi-center, open-label, randomized controlled trial, with a 2 × 2 partial factorial design to evaluate whether early CRRT support and beta-blockade will reduce 30-day mortality in a broad range of ECMO patients admitted to hospitals in China from July 2018. In arm A, patients are randomized to early CRRT within 24 h of initiation of ECMO or to control, regardless of whether there is a conventional indication, or to the control where patients only receive CRRT according to a conventional indication. In arm B, patients are randomized to the intravenous esmolol group or usual care. The trial design and protocol adhere to the Recommendations for Interventional Trials (SPIRIT) criteria [19] (Table 1) and checklist in Table 5 in Appendix. The study is planned to enroll patients from 10 centers in China (see Table 5 in Appendix for the hospital list).

**Fig. 1:**
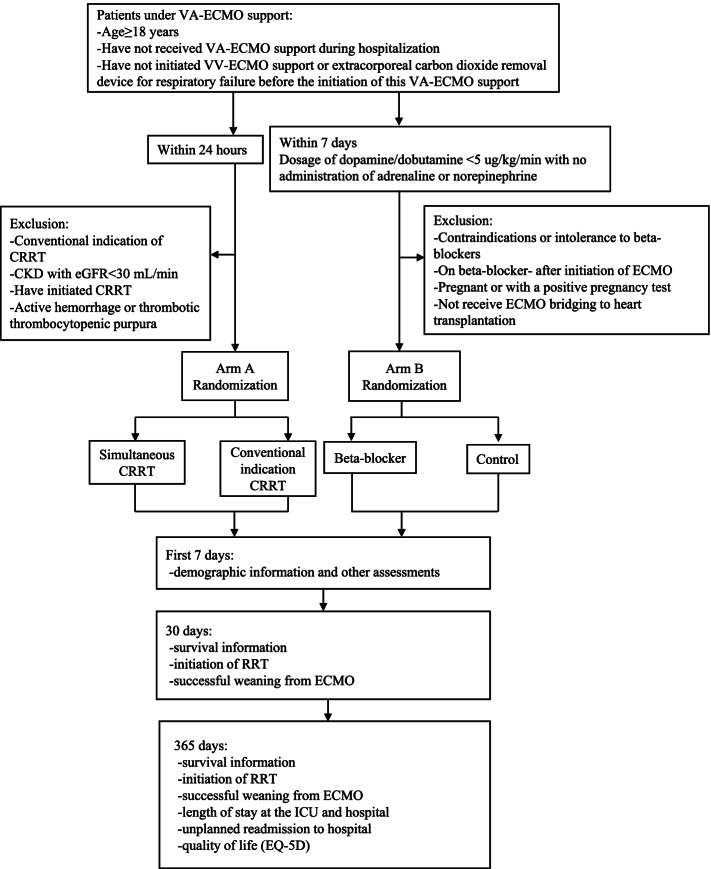
Study design. CKD, chronic kidney disease; CRRT, continuous renal replacement therapy; ECMO, extracorporeal membrane oxygenation; ICU, intensive care unit; VA-ECMO, veno-arterial extracorporeal membrane oxygenation

**Table 1:** Study period

	CRRT screening	CRRT randomization	Beta-blocker screening	Beta-blocker randomization	Days post-randomization			
					Day 3	Day 7	Day 30	Day 365
Window for time for evaluation							± 5 days	± 35 days
Visit number		1		1	2	3	4	5
Informed consent		×		×				
Demographic data	×		×					
Physical measures	×	×	×	×				
Medical history		×		×				
Concomitant medications	×	×	×	×	×	×	×	×
Physical examinations^a^		×		×				
Vital signs^b^		×		×	×	×		
Complete blood count^c^		×		×	×	×		
Arterial blood gas^d^		×		×	×	×		
Biochemistry items^e^	×		×		×	×		
Coagulation indexes^f^		×		×	×	×		
UCG^g^		×		×	×	×		
Intake and output volume in 24 h					×			
Medication, type, and dose^h^		×		×	×	×	×	×
Mechanical ventilation and mode		×		×	×	×	×	
Left ventricular unloading method					×	×	×	
RRT	×				×	×	×	×
APACHE II score		×		×				
SOFA score		×		×				
EQ-5D score								×
SAEs					×	×	×:	×

^a^Physical examinations: height (cm) and weight (kg)

^b^Vital signs: blood pressure (mmHg), pulse (beats/min), heart rate (beats/min), temperature (°C), and respiratory (/min)

^c^Complete blood count: white blood cell count (10^3^/mm^3^), hemoglobin (g/dL), and platelet count (10^9^/L)

^d^Arterial blood gas: PH, PaO_2_ (mmHg), PaCO_2_ (mmHg), K^+^ (mmol/L), Na^+^ (mmol/L), hematocrit (%), and lactic acid (mmol/L)

^e^Biochemistry items: creatine (μmol/L) and total bilirubin (μmol/L)

^f^Coagulation indexes: PT (s) and APTT (s)

^g^UCG: left ventricular ejection fraction (%), left ventricular end-diastolic diameter (cm), moderate and above pulmonary hypertension, and moderate and above tricuspid regurgitation

^h^Medication: dopamine, dobutamine, epinephrine, norepinephrine, vasopressin, pituitrin, milrinone, and beta blockers

### Eligibility

The broad inclusion criteria are used for both arms, whereby adult patients who have received ECMO for any reason within 24 h and 7 days, for arms A and arm B, respectively, are eligible (Tables 2 and 3). Patients are excluded from arms A and B if they had a definite indication or contraindication to either CRRT or beta-blockers, respectively. As ECMO is often initiated by a specialist team to rescue and transfer patients to larger hospitals in China, a timeframe of 24 h after ECMO implantation was used for early CRRT in arm A, while a relatively stable hemodynamic status (dopamine/dobutamine < 5 μg/kg/min, with no administration of adrenaline or norepinephrine) and within 7 days after initiation of ECMO was required for eligibility into arm B. Identification of potentially eligible patients is the responsibility of the ECMO team in participating centers. After ECMO implantation or when the patient is transferred to the participating center, eligibility for study arm A will be assessed. Prior to 7 days after ECMO implantation, when patients are stable on ECMO, eligibility for study arm B will be assessed.

**Table 2:** Inclusion and exclusion criteria for study A

Inclusion criteria	
Patients receiving VA-ECMO for any reason within 24 h	
Provision of informed consent	
Exclusion criteria	
Age < 18 years	
Receiving ECMO bridging to heart transplantation	
With convention indication of CRRT: AKI prior to enrollment caused by any reason, at least one of the following criteria is met:	
Severe hyperkalemia (> 6.5 mmol/L)	
Metabolic acidosis (pH < 7.2)	
Pulmonary edema	
Blood urea nitrogen level > 112 mg/dL	
Oliguria (urine output < 200 mL/12 h) for more than 72 h	
CKD, with estimated GFR<30 mL/min	
Have already initiated CRRT	
Active hemorrhage/thrombotic thrombocytopenic purpura	
Receiving ECMO again during hospitalization or respiratory failure has already initiated VV-ECMO or extracorporeal carbon dioxide removal device before the initiation of VA-ECMO of this time	

*AKI* chronic kidney disease, *CKD* chronic kidney disease, *CRRT* continuous renal replacement therapy, *ECMO* extracorporeal membrane oxygenation, *GFR* glomerular filtration rate, *VA-ECMO* veno-arterial extracorporeal membrane oxygenation, *VV-ECMO* veno-venous extracorporeal membrane oxygenation

**Table 3:** Inclusion and exclusion criteria for study B

Inclusion criteria	
Patients receiving VA-ECMO for any reason	
Dopamine/dobutamine < 5 μg/kg/min, no administration of adrenaline or norepinephrine	
Within 7 days after initiation of VA-ECMO	
Exclusion criteria	
Age < 18 years	
Receiving ECMO bridging to heart transplantation	
Contraindications or intolerance to beta-blockers	
Moderate or severe bronchial asthma attack or history of bronchial asthma	
Sinus bradycardia (heart rate < 60 bpm)	
Type II second-degree or third-degree AVB	
Allergy to esmolol	
Receiving ECMO again during hospitalization or respiratory failure has already initiated VV-ECMO or extracorporeal carbon dioxide removal device before the initiation of VA-ECMO of this time	
Have been on beta-blocker treatment after initiation of ECMO	
Women at child-bearing age, pregnant or positive pregnancy test	

*ECMO* extracorporeal membrane oxygenation, *AVB* atrioventricular block, *VA-ECMO* veno-arterial extracorporeal membrane oxygenation, *VV-ECMO* veno-venous extracorporeal membrane oxygenation

### Randomization

All eligible patients are centrally randomized in a 1:1 ratio to either early CRRT or conventional indication CRRT, and/or beta-blocker group or usual care (control) groups, via a computer-generated randomization schedule, stratified by age (< 65 vs. ≥ 65 years) and implantation of ECMO for extracorporeal cardiopulmonary resuscitation (yes vs no). The sequence of randomization is generated by an independent statistician, using SAS version 9.4. Investigators use a cellphone application to confirm eligibility and obtain the randomized treatment allocation.

### Interventions

Patients assigned to early CRRT should have it applied concurrently with ECMO treatment and continued for ≥ 12 h. Those assigned to standard therapy should receive CRRT according to the 2012 Kidney Disease: Improving Global Outcomes (KDIGO) Clinical Practice Guideline for AKI stage 3 and have at least one of the following criteria met: severe hyperkalemia (> 6.5 mmol/L), metabolic acidosis (pH < 7.2), pulmonary edema unresponsive to diuretic therapy, requiring oxygen flow rate of > 5L/min to maintain an oxygen saturation (SpO_2_) of > 95% or requiring a forced inspired oxygen concentration (FiO_2_) of > 50% on ventilation, blood urea nitrogen level > 112 mg/dL, or oliguria (urine output < 200 mL per 12 h) for > 72 h. CRRT can be initiated or discontinued according to the attending clinician’s decision in managing the patient.

In arm B, patients assigned to the intervention group are to receive a continuous infusion of esmolol on top of conventional management, commencing at 25 mg/h and increasing by 25 mg/h every 20 min until the heart rate is reduced to 75 ± 5 bpm or an upper dose limit of 2000 mg/h is reached. Esmolol infusion should be continued to maintain the heart rate threshold or according to the clinician’s discretion until discharge from the intensive care unit (ICU) (or death, if sooner). Oral beta-blockers should be introduced before esmolol is withdrawn. Esmolol can be transiently stopped or completely withdrawn should a patient develops third-degree atrial-ventricular block, bradycardia (< 60 bpm), extreme left ventricular systolic dysfunction, aortic valve dysfunction, or severe cardiogenic pulmonary edema. Patients in the control group will not receive any type of beta-blockers, unless the clinician considers there is a strong indication. Concomitant diseases will be treated following the current guidelines.

### Outcomes

The proportion of all-cause mortality at 30 days will be compared between the intervention and control groups. Survival data will be collected through telephone follow-up. Secondary outcomes are all-cause mortality at 365 days; use of long-term RRT; success in weaning from ECMO, defined as survival after 24 h from weaning; health-related quality of life according to scores on the EuroQoL (EQ-5D-5L) questionnaire at 365 days; length of stay at ICU and hospital; unplanned hospital readmission; and separately on cardiac and non-cardiac death.

Severe adverse events (SAEs), such as major bleeding, cardiac arrhythmias, ventilator-associated pneumonia, bloodstream infection, surgical site infection, limb ischemia from any cause, and ischemic or hematologic stroke will be collected systemically. Any other SAEs determined by the physicians through spontaneous reporting (Table 4 outlines SAE definitions). The publication will detail each documented SAE. All the SAEs will be coded through MedDRA.

**Table 4:** Definition of SAEs

The SAEs include but are not limited to	
Major bleeding	
Bleeding requires transfusion for > 2 units	
Severe arrhythmias	
Type II second-degree AVB or third-degree AVB	
Sustained ventricular tachycardia (> 30 s)	
Ventricular fibrillation	
Ventilator associated pneumonia, which needs to meet all the criteria below:	
At least 48 h after endotracheal intubation	
Chest X-ray showing sustained or worsening shadowing (infiltrates or consolidations)	
Signs of pulmonary consolidation and/or crackles on auscultation, at least meet one of the following criteria:	
a) White blood cell count > 10 × 10^9^/L or < 4 × 10^9^/L	
b) Temperature > 37.5 °C, purulent secretions	
c) Positive cultures obtained directly from bronchial secretions	
Bloodstream infection	
Positive cultures obtained from the peripheral blood	
SSI	
Purulent drainage in the operated region with pain or tenderness, localized swelling, redness, and heat or fever, requiring operation, including superficial incisional wound SSI, deep incisional wound SSI, and organ/space SSI	
Limb ischemia from any cause	
Physical examination demonstrating pain, pallor, pulseless, cold, and motor or sensor deficit	
Requiring decannulation of ECMO catheter or surgical intervention	
Stroke	
New onset abnormal neurological signs and symptoms last for at least 24 h with radiographic evidence	
Any other severe adverse events determined by the physicians	

*AVB* atrioventricular block, *ECMO* extracorporeal membrane oxygenation, *SAE* serious adverse events, *SSI* surgical site infection

### Data management

Data are collected on patient demography, medical history, concomitant therapy, duration of CRRT, and dose and duration of esmolol. Vital and health status are assessed at the time of weaning from ECMO, discharged from ICU and hospital, unplanned hospital readmission, EQ-5D scores, and SAEs, during follow-up via telephone, face-to-face, or remote medical consultation over 365 days. To improve monitoring adherence, the clinician will take time to explain about the need for follow-up surveillance and encourage the participants to undergo routine examinations. Data are entered into a secure password-protected electronic data capture system and checked for quality by research staff. All queries are listed for content and raised and resolved dates. All study records required by the coordinating center at the Heart Health Research Center (HHRC) and applicable regulatory bodies are maintained for 15 years. The confidentiality of all participant information must be protected at the clinical sites and the coordinating center. Paper records and computer files must be appropriately safeguarded from unauthorized access.

### Sample size

Sample size calculation is performed using the software PASS 15.0.5 based on the following assumption: a 70% 30-day mortality in the control group and a 20% relative risk reduction for each intervention (early CRRT and beta-blocker). The estimate of the intervention’s effect size was based on clinical expertise and the clinical significance of an intervention, no loss of follow-up or crossover, and 5% type I error and 20% type II error. We assume no interaction between interventions, as the overlap of the time of interventions would be limited and no carry-on effect expected. A total of 496 patients (248 per group) are required for each arm.

To achieve adequate participant enrolment to reach the target sample size, the study will be conducted at 10 high-volume centers in China. A smartphone application has been developed to facilitate randomization at the emergency room.

### Statistical analysis

All analyses will be conducted according to the intention-to-treat principle by blinded statisticians. All patients will be analyzed in accordance with the results of randomization, regardless of whether they received the prescribed therapy. Baseline characteristics between the groups will be reported as frequencies and percentages for categorical variables and as means and standard deviations (SD) or medians and interquartile ranges (IQR) for continuous variables. The primary outcome will be compared between the groups in a log-rank test. Other efficacy and safety endpoints will be reported with the *t* test or Wilcoxon test, and the chi-square test for continuous and categorial variables, respectively, as appropriate. All tests are two-sided, and the nominal level of *α* will be 5%.

In the subgroup analysis, age and ECPR will be defined by the presence or absence of a pre-randomization variable and the primary outcome as in the main analysis. The main analysis for each subgroup will be an unadjusted test of interaction in a logistic model to determine whether the effect of treatment differs significantly across categories. The missing values will not be imputed unless substantial. The number of observations used in the analysis will be reported.

### Monitoring

The data monitoring committee (DMC) consists of a critical care physician, nephrologist, and cardiovascular expert, all experienced in clinical research. The principal investigator will report the process of the trial, quality of data collected, adverse events, and protocol deviation and violation to the DMC. The DMC will meet every 6 months during the conduction of the trial. No interim analysis for efficiency is planned as the assumptions in sample size calculation are less likely to be changed.

An investigator meeting is held annually to communicate the progress, quality control, and approaches to improve recruitment and address any quality of data issues.

### Trial status

There have been 89 patients enrolled at 10 hospitals in study A, and the enrollment is still ongoing. However, study B was stopped in August 2019 in the absence of any patients being enrolled. Although we aimed to test the hypothesis that beta-blockers would protect the myocardium in ECMO patients, we found this too challenging to undertake as these patients have low BP, which is a contraindication to such treatment and raised concerns of harm among investigators. Moreover, the short time window for enrollment was another barrier to recruitment, and by the time a patient is stable and without vasopressors, it is near the time to wean them off ECMO. Consequently, we decided to close recruitment into arm B. During the COVID pandemic, patient enrollment has become more challenging. A review of the feasibility of the trial will be made by the Steering Committee at the end of 2022.

## Discussion

Being one of the most seriously ill groups in clinical practice, with high mortality and requiring heavy use of critical care resources, there are considerable challenges to generate reliable evidence to guide the management of ECMO patients. Our ELITE trial attempts to address two important clinical questions with strong pathophysiological mechanisms.

A multicenter retrospective cohort study has shown that FO is common in a pediatric population (peak FO ≥ 10% in 84.8%, ≥ 20% in 67.2%, and ≥ 50% in 29%) [20] where FO has consistently been shown to be associated with adverse outcomes [21–23], while a meta-analysis of observational studies has shown lower mortality in those who receive CRRT [24]. These data suggest that early initiation of CRRT to avoid FO in the context of ECMO may be beneficial. The Kidney Interventions During Membrane Oxygenation (KIDMO) study showed that CRRT use was 43%, 16%, and 35% of patients for FO, for FO prevention, and for AKI, respectively, after ECMO [25]. However, there has not been a randomized trial to support these approaches. So, we designed this ELITE trial. As the closing of arm B, the question of whether beta-blocker is efficient in patients with ECMO remains unaddressed, and more effects should be made.

Other limitations of our trial include the lack of blinding, and for practical reasons, recruitment has been slow due to the small number of patients receiving ECMO despite the expectation of a least 10 cases treated annually across our network of 30 ICUs in China. We did not stratify our randomization by ECMO centers but will perform a subgroup analysis to explore the effect between small and large centers.

In summary, the ELITE trial is the first randomized controlled trial of critically ill patients receiving ECMO support, powered to test the effects of early CRRT versus conventional timing of CRRT on 30-day mortality. The results should inform the management of this important patient group.

## Data Availability

The datasets used and analyzed during the current study are available from the corresponding authors on reasonable request.

## Appendix

Please insert Table 5 here

**Table 5:** ELITE study centers

Centers	Location
The Secondary Affiliated Hospital of Zhengzhou University	Zhengzhou, Henan province
The First Affiliated Hospital of Zhengzhou University	Zhengzhou, Henan province
The Second Hospital of Jilin University	Changchun, Jilin province
The People’s Hospital of Guangxi Zhuang Autonomous Region	Nanning, Guangxi Zhuang Autonomous Region
Chinese PLA General Hospital	Beijing
Beijing Anzhen Hospital	Beijing
Zhongshan City People’s Hospital	Zhongshan, Guangdong province
Sichuan Provincial People’s Hospital	Chengdu, Sichuan province
Taizhou Hospital of Zhejiang Province	Taizhou, Zhejiang province
Zhongshan Hospital Affiliated to Fudan University	Shanghai
